# A systematic High-Content Screening microscopy approach reveals key roles for Rab33b, OATL1 and Myo6 in nanoparticle trafficking in HeLa cells

**DOI:** 10.1038/srep28865

**Published:** 2016-07-04

**Authors:** Angela Panarella, Mariana G. Bexiga, George Galea, Elaine D. O’ Neill, Anna Salvati, Kenneth A. Dawson, Jeremy C. Simpson

**Affiliations:** 1 School of Biology and Environmental Science, University College Dublin, Belfield, Dublin 4, Ireland; 2 Conway Institute for Biomolecular and Biomedical Research, University College Dublin, Belfield, Dublin 4, Ireland; 3 Centre for BioNano Interactions, School of Chemistry and Chemical Biology, University College Dublin, Belfield, Dublin 4, Ireland

## Abstract

Synthetic nanoparticles are promising tools for imaging and drug delivery; however the molecular details of cellular internalization and trafficking await full characterization. Current knowledge suggests that following endocytosis most nanoparticles pass from endosomes to lysosomes. In order to design effective drug delivery strategies that can use the endocytic pathway, or by-pass lysosomal accumulation, a comprehensive understanding of nanoparticle uptake and trafficking mechanisms is therefore fundamental. Here we describe and apply an RNA interference-based high-content screening microscopy strategy to assess the intracellular trafficking of fluorescently-labeled polystyrene nanoparticles in HeLa cells. We screened a total of 408 genes involved in cytoskeleton and membrane function, revealing roles for myosin VI, Rab33b and OATL1 in this process. This work provides the first systematic large-scale quantitative assessment of the proteins responsible for nanoparticle trafficking in cells, paving the way for subsequent genome-wide studies.

Since their first appearance in the second half of the previous century, nanoparticles (NPs) have presented an opportunity for maximizing specificity and efficacy of diagnostic and therapeutic agents^1^; however, many challenges still remain hampering their full application in clinical practice. In addition to widely-reported challenges associated with *in vivo* targeting, pharmacokinetics and toxicity^2^,^3^, our lack of understanding of the intracellular fate of nanomaterials represents another obstacle to drug efficacy. In this regard, the investigation of intracellular delivery modalities of nanomaterials is important for the development of drug delivery strategies in the heterogeneous context of tumors or inflammatory diseases in which the rate of uptake can have significant effects on drug efficacy. Even more importantly, strategies to engineer their final intracellular destination, for example escaping the degradative endo/lysosomal system, are key considerations in drug carrier design^4^. Many aspects need to be taken into account in order to study the uptake and intracellular trafficking of nanomaterials, such as their structural and physicochemical characteristics^5^, the biological molecules coating the nanomaterial (the corona^5^,^6^), as well as specific cell-type differences in the endocytic machinery^7^.

Chemical inhibitors have traditionally been used to elucidate the molecular mechanism by which NPs enter and traffic through cells^8^, but such tools are crude, providing relatively little specificity in terms of identifying the key molecules involved^9^. By contrast, RNA interference (RNAi) technologies, such as those utilizing small interfering RNAs (siRNAs) to selectively and systematically deplete cellular proteins, have proved to be an invaluable tool to dissect membrane trafficking events in human cells. Specific notable examples include their application to study endocytosis^10^ and secretion^11^. Furthermore, the combination of RNAi with automated high-content screening (HCS) microscopy potentially allows for the molecular dissection of cellular events with spatial and temporal resolution from single cells and across a population^12^, representing an unbiased approach for the discovery of new players in fundamental intracellular processes^13^. A small number of studies utilizing HCS microscopy to relate membrane trafficking to the efficiency of intracellular drug delivery have been reported, although these were either based on the use of libraries of chemical inhibitors^14^ or on a small number of individually selected siRNAs^15^. Here we describe and apply an RNAi-HCS approach utilizing two siRNA libraries to specifically deplete families of proteins associated with either the cytoskeleton or the endomembrane system, in order to provide the first gene-based systematic overview of the mechanism by which synthetic NP internalization and delivery to acidic cellular organelles occurs.

## Results and Discussion

**Figure 1:**
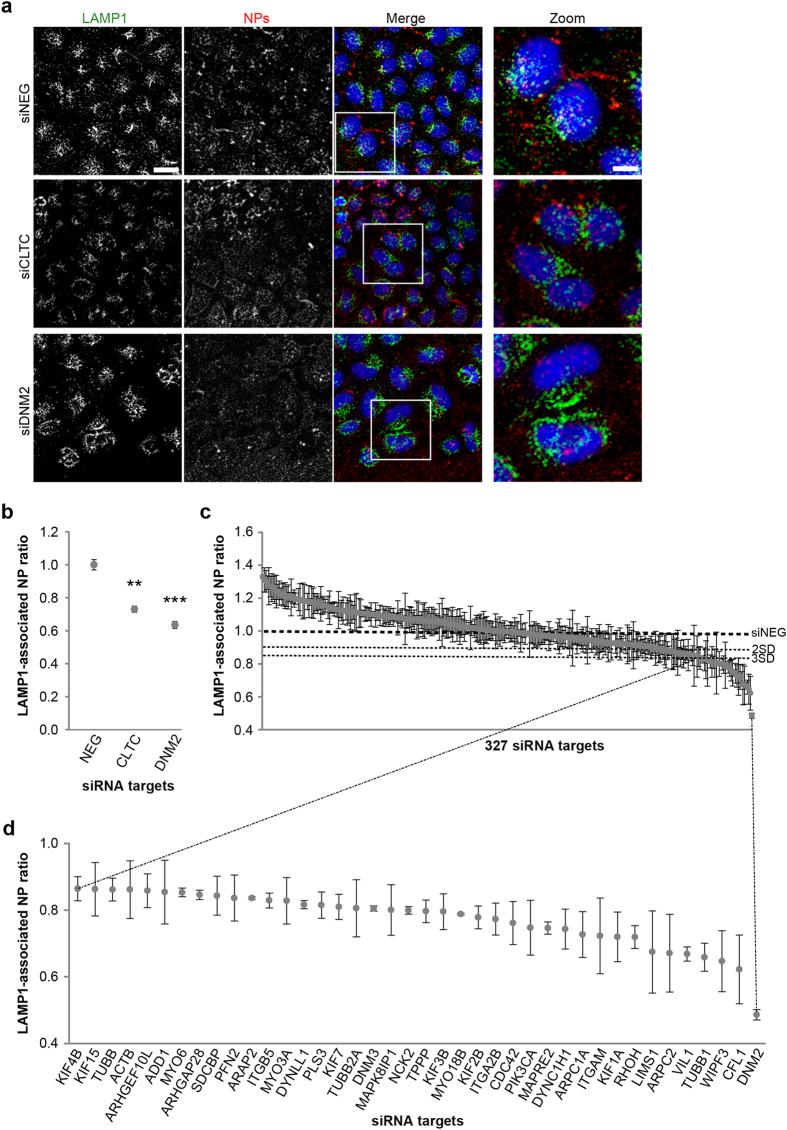
Primary RNAi screen of cytoskeleton library for NP delivery to LAMP1-positive membranes. (**a**) Example images of HeLa cells treated with non-targeting siRNA (siNEG) or targeting clathrin heavy chain (siCLTC) or dynamin2 (siDNM2) and then pulse chased with 40 nm polystyrene carboxylated NPs for 60 minutes, fixed and immunostained for LAMP1. In the merged images, nuclei are in blue, LAMP1 in green and NPs in red. Scale bars, 35 μm and 10 μm in zoom. (**b**) Graph showing LAMP1-associated NP ratio in control cells (NEG) or cells treated with siRNAs targeting CLTC or DNM2. (**c**) Graph showing LAMP1-associated NP ratio in cells treated with a library of siRNAs targeting genes associated with cytoskeleton function. (**d**) Enlargement of (**c**) showing LAMP1-associated NP ratio in cells treated with siRNAs that caused a strong reduction (3 standard deviations, SD) of NP delivery to LAMP1-positive membranes compared to siNEG-treated cells. In (**b–d**) values represent mean and s.e.m. of 3 independent experiments normalized to the value shown by cells treated with siNEG; **p-value < 0.01; ***p-value < 0.001.

Nanomaterials, and NPs in particular, are available in a wide range of materials, sizes and shapes, each of which will have unique properties. One of the most widely studied NP types are those made from polystyrene, as they are typically homogenous in size and dispersity and have been extensively characterized and utilized for both *in vitro* and *in vivo* studies^16^,^17^,^18^,^19^,^20^. Furthermore, the ability to label them with fluorescent reporters means that they are an ideal model for imaging applications^21^, potentially providing a first level of information about how NPs interact with cells. In this study we selected 40 nm carboxylated polystyrene (PS-COOH) NPs as our model, as their size falls within the range of endocytic cargoes encountered by cells and therapeutic vectors^2^ (for size and charge characterization see Supporting Information Table S1). As a read-out for our screen, we decided to quantify the portion of intracellular NPs associated with the lysosomal protein LAMP1; this value is hereafter termed the ‘LAMP1-associated NP ratio’. LAMP1 was selected as a suitable marker, as it defines the terminal organelle for NP trafficking following their internalization into cells, as reported previously^22^,^23^. This LAMP1-associated NP ratio value would represent the amount of NP signal detected in LAMP1-positive structures as a proportion of the NP signal detected within the entire cell region. For any treatment or perturbation, this value can then be normalized to the ratio observed in control cells. As a consequence, LAMP1-associated NP ratios lower than 1 would represent a reduction of NP trafficking from the cell periphery to the lysosomal compartment compared to control cells, whereas a ratio higher than 1 would represent an enhanced rate of NP delivery to this compartment. In order to demonstrate the suitability of our approach we tested the involvement of clathrin-mediated endocytosis machinery on NP uptake, as this pathway has been proposed as being significant for polystyrene NP uptake into cells^24^. In addition to depletion of the clathrin heavy chain subunit (CLTC), we also investigated the role of dynamin 2 (DNM2), a large GTPase that controls the scission of vesicles, including those coated with clathrin, internalizing from the plasma membrane^25^,^26^,^27^. HeLa cells were treated for 72 hours with negative control siRNAs (NEG) with no cognate intracellular target, or with siRNAs targeting either CLTC or DNM2, followed by 40 nm PS-COOH NP addition and immunostaining of the lysosomal protein LAMP1 (Fig. 1a). Quantitative analysis of the images revealed a significant decrease in the LAMP1-associated NP ratio compared to that seen in control cells (NEG) in both cases (Fig. 1b). Efficiency of knock-down was quantified by both qPCR and western blotting (reported in Supporting Information Figures S1 and S2); in all cases resulting in a reduction of the target mRNAs and cognate proteins by at least 80%. These experiments established that automated HCS microscopy can be used to assess the role of individual genes on NP trafficking to LAMP1-positive compartments.

In order to gain new mechanistic insight into NP uptake and intracellular trafficking we extended this approach by interrogating a custom-designed siRNA library targeting 348 genes involved in cytoskeleton organization, function and regulation. From this primary screen, of the 327 depletions that showed sufficient cell viability for analysis (see Methods for definition), 29 induced a mild reduction and 50 resulted in a strong reduction in LAMP1-associated NP intensity (Fig. 1c and Supporting Information Table S2). In order to remove imaging and analysis artifacts coming from the automated pipeline, the strongest 50 candidates were visually inspected resulting in 40 gene targets being retained (Fig. 1d). Among these, and consistent with the previous experiments, depletion of DNM2 resulted in the strongest reduction (50%) in delivery of NPs to LAMP1-positive compartments, and was therefore selected as a positive control in subsequent experiments. Of the 39 remaining candidate genes, 7 were found to be kinesin motor subunits, 2 were cytoplasmic dynein complex subunits, 3 were myosin isoforms, and the remainder were associated with actin and tubulin cytoskeleton regulation.

**Figure 2:**
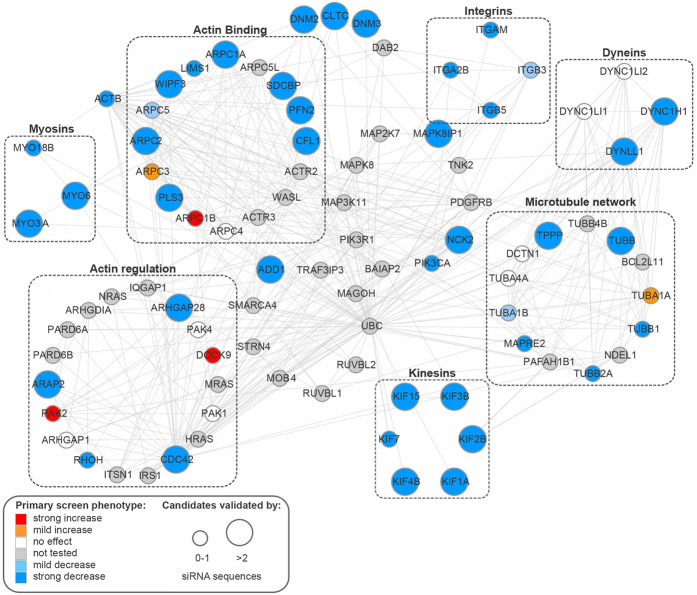
Validation of cytoskeleton screen candidates and prediction of interactors. Validated candidate genes were subjected to STRING analysis in order to predict other relevant molecules associated with NP uptake and trafficking. Blue nodes indicate siRNA depletions inducing a strong decrease in the LAMP1-associated NP ratio, light blue indicates a mild decrease. Red nodes indicate siRNA depletions inducing a strong increase in the LAMP1-associated NP ratio, orange nodes indicate a mild increase. Gray and white nodes indicate genes not tested and treatments with no effect, respectively. Large nodes represent phenotypes validated by at least two independent siRNAs.

All the candidate genes were interrogated by the STRING database in order to identify other possible relevant molecules associated with NP uptake and trafficking, thereby allowing us to enrich the list of interactors for each of the families to which the candidate genes could be associated (Fig. 2). This highlighted 13 targets already tested in the primary screen, but which did not induce any decrease in NP uptake (small white, red or orange circles), and 3 that induced a mild phenotype (ARPC5, TUBA1B, ITGB3, depicted as light blue small circles). It also allowed the identification of 33 additional proteins with a potential role in NP uptake and trafficking (small gray circles). Further experiments will be needed to validate whether these additional nodes, which are part of the predicted network, also play a role in NP trafficking. Of particular interest is the protein ubiquitin (UBC), which from the STRING analysis localizes at the crossroads of both the actin and microtubule cytoskeletons, and which is known to be an important sorting signal for degradation in endocytosis^28^. In order to validate the candidate genes identified in the primary screen, cells were next separately transfected with three independent siRNAs against each target. To further improve cell viability, these validation experiments were performed after a 48 hour transfection. Of the 39 candidate genes, 25 were validated with at least two individual siRNAs (depicted as large nodes in Fig. 2, full list of results in Supporting Information Table S3).

For further validation, selected members of each family identified in the siRNA screen were analyzed by qPCR (Figure S3 in Supporting Information). Together these experiments revealed, for the first time, sets of proteins influencing NP uptake and trafficking. Of particular note was the identification of several motor protein subunits (DYNC1H1, KIF15, MYO6) and proteins associated with actin and microtubule structure and remodeling (ARPC2, CDC42, CFL1, PLS3, TUBB) (Fig. 2). We also observed the involvement of another dynamin variant, DNM3, but not of the neuronal-specific DNM1 (Fig. 1d). Combined depletion of both DNM2 and DNM3 showed an additive, albeit not statistically significant, decrease of NP trafficking to LAMP1-positive membranes (Figure S4 in Supporting Information). Taken together, these results suggest that the transfer of NPs through the endomembrane system is an active and highly regulated process, likely involving several membrane intermediates prior to lysosome delivery.

**Figure 3:**
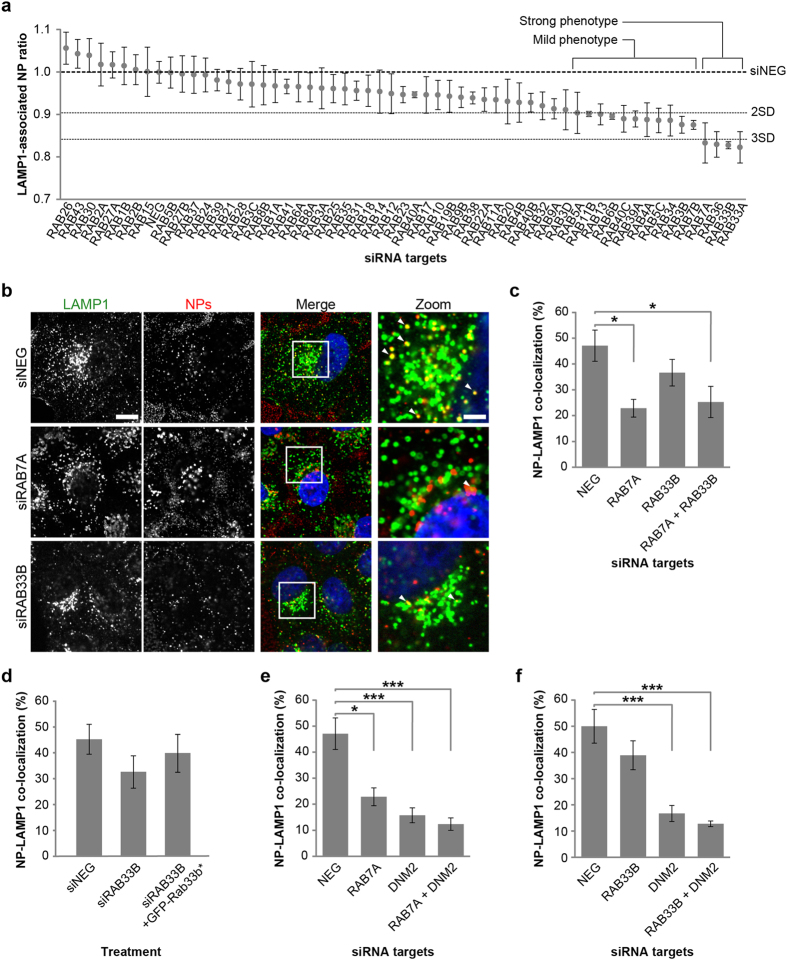
Primary RNAi screen of the Rab library for NP delivery to LAMP1-positive membranes and validation of candidates. (**a**) Graph showing LAMP1-associated NP ratio in cells treated with a library of siRNAs targeting 58 RAB genes. (**b**) Example confocal images of HeLa cells transfected with non-targeting siRNA (siNEG) or targeting RAB7A or RAB33B and then pulse-chased with 40 nm PS-COOH NPs for 60 minutes, fixed and immunostained for LAMP1. In the merged images, nuclei are in blue, LAMP1 in green and NPs in red. Arrowheads indicate co-localizing structures. Scale bars, 20 μm and 6 μm in zoom. (**c,e,f**) Graphs showing percentage of NPs co-localizing with LAMP1-positive membranes in control cells (NEG) or cells treated with siRNAs targeting RAB7A, RAB33B, DNM2 or combinations as indicated. (**d**) Graph showing the percentage of NPs co-localizing with LAMP1-positive membranes in control cells (NEG), or treated with RAB33B siRNA and then either left untreated (siRAB33B) or transfected with an siRNA-resistant GFP-RAB33B construct (Rab33b*). In (**a**) values represent mean and s.e.m. of 3 independent experiments normalized to the value shown by cells treated with siNEG. In (**c–f**) values represent mean and s.e.m. of 3 independent experiments; *p-value < 0.05; ***p-value < 0.001.

In order to further probe this, and add context to the candidates identified so far, a second siRNA library was designed, but now targeting the Rab small GTPases, a subgroup of the Ras superfamily widely distributed throughout the cell^29^. Rab proteins show a high degree of organelle-specificity and we have previously used live-cell imaging to show that PS-COOH NPs pass through membranes decorated with Rab5, Rab9 and Rab7^30^. Of the 58 Rabs targeted in the RNAi approach described here, depletion of 11 of them resulted in a mild decrease of LAMP1-positive organelle delivery, while 4 displayed a strong decrease (Fig. 3a and Supporting Information Table S4).

Of the 11 siRNA treatments that induced a mild phenotype, 9 of the targets were Rab proteins associated with the endocytic pathway (Rab4a, Rab5a, Rab5c, Rab7b, Rab11b, Rab13, Rab34, Rab39a, Rab40c). Of particular note was our observation that depletion of Rab5a resulted in a reduction in delivery of NPs to lysosomes, as we have previously reported that PS-COOH NPs transit through Rab5a-positive endosomes *en route* to more acidic organelles^30^. The 4 target genes showing the strongest phenotype (RAB7A, RAB33A, RAB33B, RAB36) were tested by qPCR analysis using two independent siRNA sequences (Supporting Information Table S5 and Figure S5). Greater than 65% reduction in mRNA levels for RAB7A and RAB33B was observed, but we were unable to reliably detect RAB33A and RAB36 transcripts, even in control cells, possibly suggesting off-target effects of these siRNAs, and therefore these targets were not considered further, allowing us to concentrate on the effects observed on depletion of RAB7A and RAB33B. We repeated our NP uptake experiments in cells depleted for each of these mRNA targets, now using confocal images, and carried out precise quantitative co-localization analysis using the Rank Weighted Co-localization (RWC) coefficient^31^ as a measure of the co-occurrence of NP signal and LAMP1 (Fig. 3b). The measurements obtained were similar to those seen in the automated analysis, clearly showing that NP delivery to lysosomes is affected by the levels of these two proteins (Fig. 3c). Western blot analysis confirmed the successful depletion of Rab33b protein in cells treated with siRNAs targeting RAB33B mRNA (Supporting Information Figure S6). Visual examination of a number of the images revealed occasional changes in cellular distribution of the lysosome population. Therefore, to confirm that any significant changes in the LAMP1-associated NP ratios detected across the screens were not simply a consequence of gross changes in lysosome organization, we quantified the mean LAMP1 fluorescence intensity per cell for every siRNA treatment (Supporting Information Tables S2 and S4). This analysis revealed no significant variation in LAMP1 levels in any of the siRNA treatments.

**Figure 4:**
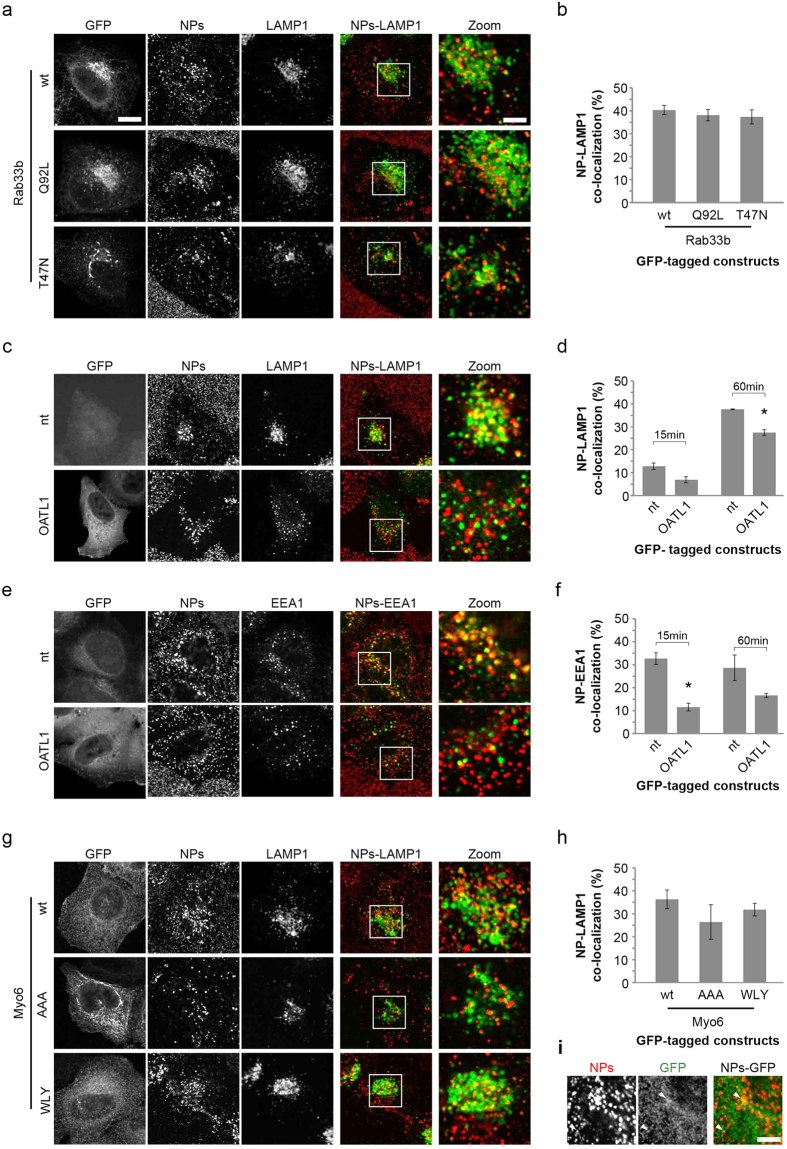
Secondary analysis of validated gene candidates for NP delivery to LAMP1- or EEA1- positive membranes. (**a**) Example images of HeLa cells transfected with plasmid DNA encoding GFP-tagged Rab33b wild-type (wt), active (Q92L) or inactive (T47N) variants and then pulse-chased with 40 nm PS-COOH NPs (in red) for 60 minutes, fixed and immunostained for LAMP1 (in green). Scale bars, 20 μm and 6 μm in zoom. (**b**) Graph showing percentage of NPs co-localizing with LAMP1-positive membranes in cells expressing GFP-tagged wild-type or point mutants of Rab33b. (**c**) Example images of HeLa cells transfected with plasmid DNA encoding OATL1 or non-transfected (nt) and pulse chased with 40 nm PS-COOH NPs (in red) for 60 minutes, fixed and immunostained for LAMP1 (in green). (**d**) Graph showing percentage of NPs co-localizing with LAMP1 in cells expressing GFP-tagged OATL1 or non-transfected (nt). (**e**) Example images of HeLa cells transfected with plasmid DNA encoding GFP-tagged OATL1 or non-transfected (nt) and pulse-chased with 40 nm PS-COOH NPs (in red) for 15 minutes, fixed and immunostained for EEA1 (in green). (**f**) Graph showing percentage of NPs co-localizing with EEA1 in cells expressing GFP-tagged OATL1 or non-transfected (nt). (**g**) Example images of HeLa cells transfected with plasmid DNA encoding GFP-Myo6 or two mutated forms and pulse chased with 40 nm PS-COOH NPs (in red) for 60 minutes, fixed and immunostained for LAMP1 (in green). (**h**) Graph showing percentage of NPs co-localizing with LAMP1 in cells expressing GFP-tagged Myo6 or two mutated forms. (**i**) Zoom of (**g**) showing partial co-localization of NP and Myo6-wt, arrowheads indicate co-localizing structures. In (**b,d,f,h**) values represent mean and s.e.m. of 3 independent experiments; *p-value < 0.05.

In order to further understand the NP phenotype observed on depletion of Rab33b, we incubated cells with LysoTracker Red as a general marker of lysosomes, and measured its fluorescence intensity and spot number per cell. These experiments revealed no significant change in either lysosome intensity or number, in cells depleted for Rab33b when compared to control cells, thereby suggesting that the observed reduction in LAMP1-associated NP ratio and decreased co-localization between these two markers on Rab33b depletion is a direct consequence of a trafficking defect (Supporting Information Figure S7). To further confirm the specificity of the Rab33b phenotype, we next prepared a DNA expression construct encoding GFP-fused wild-type Rab33b, but which contained a number of silent nucleotide changes which would make the mRNA resistant to RAB33B siRNA treatment. Overexpression of this construct in cells also incubated with the siRNAs targeting RAB33B was able to partially recover the levels of co-localization of NPs with LAMP1 to that seen in control cells, strongly supporting the notion that Rab33b is a direct regulator of NP delivery to lysosomes (Fig. 3d). Interestingly, the combined depletion of DNM2 with either RAB7A or RAB33B in both cases resulted in an additive (although not statistically significant) effect in terms of a further reduction of delivery of NPs to LAMP1-positive organelles (Fig. 3c,e,f), consistent with distinct roles for DNM2 at the cell surface and the two Rabs on internal membranes. Since these results supporting a role for the late endosomal Rab7a in NP trafficking were in good agreement with our previous observations^30^, we decided to further investigate Rab33b, as this has never previously been linked to NP internalization or trafficking. Rab33b has been described as a medial Golgi complex protein involved in Golgi-to-ER retrograde traffic^32^, although it has also been suggested to play a role in autophagy^33^, the intracellular degradative pathway responsible for nutrient recycling and clearance of protein aggregates, damaged organelles and microbes. Rab33b has been linked to autophagosome formation, facilitating elongation and expansion of the phagophore membrane^34^. Like all Rab proteins, Rab33b acts as a molecular switch, cycling between a GDP-bound (inactive) form and a GTP-bound (active) form, in turn regulating membrane traffic function. We therefore examined the effect of overexpression of GFP-tagged Rab33b GDP/GTP-restricted forms on the trafficking of NPs to LAMP1-positive organelles by quantitative co-localization analysis in manually acquired confocal microscopy images. Surprisingly, we were unable to observe any significant change in the intracellular distribution of NPs (Fig. 4a) or alteration in the NP-LAMP1 co-localization coefficient in the presence of any of the Rab33b mutants compared to control cells (Fig. 4b).

Although the use of such mutants is well-established in terms of probing Rab function, one drawback is that they are typically used in cells containing a background of endogenous wild-type protein, thereby limiting any phenotypic effect. We, then, decided to observe NP trafficking after overexpression of GFP-OATL1, a previously described GTPase activating protein (GAP), and an established key regulator of Rab33b^35^. In cells overexpressing GFP-OATL1, internalized NPs showed a more peripheral distribution (Fig. 4c) and a corresponding decrease in co-localization with LAMP1 when compared to non-transfected cells was observed at both early and late internalization time-points (Fig. 4d). When we examined NP trafficking through EEA1-positive early endocytic compartments in cells overexpressing GFP-OATL1, we also observed a strong reduction in NP association with these organelles (Fig. 4e,f). These results strongly suggest a role for Rab33b and OATL1 in the trafficking of NPs through early endocytic membranes to late endosomes/lysosomes. Combining this with our previous observations^22^,^30^,^36^ and taking into account the recently reported role for Rab7a in autophagy^37^, our data also suggest critical roles for Rab33b and Rab7a in delivery of NPs to autophagy-related membranes. These results provide further context for the cytoskeleton-associated regulators identified earlier in this study, specifically the molecular motors involved in the recruitment of autophagic complexes and membranes. Myosin VI (Myo6) is a minus-end-directed motor protein that has established roles in the tethering and sorting of cargo during endocytosis, exocytosis and endosomal sorting, with an associated role in autophagy^38^, hence making it a likely candidate to play a role in uptake of large molecules such as NPs. Non-polarized cells express the myosin VI non insert isoform, which is associated with a non-mature subpopulation of early endosomes that lack clathrin and EEA1, but are decorated with Rab5 and the multifunctional adaptor protein APPL1^39^. The interaction between APPL1 and myosin VI is facilitated by another adaptor protein GIPC, which associates with the RRL amino-acid motif in the myosin VI tail region^40^. Another amino-acid motif, WWL, allows interaction between myosin VI and the adaptor TOM1, which targets myosin VI to early endosomes for maturation of autophagosomes or fusion with lysosomes^41^. Overexpression of two mutated forms of myosin VI (WWY to WLY or RRL to AAA) induced a mild although not statistically significant decrease in NP-LAMP1 co-localization compared to cells transfected with the wild-type form (Fig. 4g,h). Although myosin VI cannot easily be visualized in discrete punctate structures, in some areas of the cell a partial overlap of NP and myosin VI signals was observed (Fig. 4i). Since the RRL motif is also responsible for the interaction between myosin VI and Optineurin, T6BP and NDP52, three cargo-selective receptors for autophagy^41^,^42^,^43^, our data support a role for myosin VI in the internalization and lysosomal localization of NPs possibly via an autophagy pathway.

Although the work presented here focuses on the intracellular trafficking mechanisms of a single NP type, it nevertheless lays the foundations for future studies that could consider the effects of particle size, material, shape, and modification. In this regard, the technological approach that we describe is highly quantitative and can be readily adapted as required. Although polystyrene is not a material of high clinical value, the NPs that we have used show homogenous size and dispersity, and display little acute toxicity^36^, and as such they represent a paradigm for evaluating at a molecular level how nanomaterials interact with cells.

## Conclusions

In this work we present methodology and application of the first systematic assessment of the molecular machinery involved in the intracellular trafficking of NPs. Our findings reveal central roles for a variety of molecule classes, including early endocytic machinery (clathrin heavy chain and dynamin 2), late endosomal regulators (Rab7a), as well as autophagy-related proteins (Rab33b, OATL1 and myosin VI). The approach used here identifies several proteins involved in the intracellular processing of NPs, adding molecular detail to the bio-nano interaction environment and establishing the foundations for the design of more efficient intracellular drug delivery strategies.

## Materials and Methods

### Cell culture

HeLa cells were cultured at 37 °C in Dulbecco’s modified Eagle medium containing 1 g/l D-glucose and 1 mM sodium pyruvate (DMEM, Gibco/Life Technologies) supplemented with 10% heat inactivated fetal bovine serum (FBS, PAA/GE Healthcare) and 1% glutamine (Sigma-Aldrich).

### Nanoparticle characterization

Fluorescently-labeled polystyrene carboxylated NPs (PS-COOH, red, 40 nm, Invitrogen) were used. Freshly prepared nanoparticle dispersions (concentration of 100 μg/ml) were characterized in water, phosphate buffered saline (PBS), serum-free cell culture medium (sf-DMEM) or 10% serum complemented (c-DMEM) at 25 °C or at 37 °C. NP size (hydrodynamic diameter) and zeta-potential (related to surface charge) were measured using a Zetasizer Nano ZS90 (Malvern Instruments) and measurements are reported in Supporting Information Table S1.

### siRNA solid phase reverse transfection

For the primary screen, two custom-designed libraries targeting cytoskeletal proteins (Ambion/Life Technologies) and proteins of the Rab family (siGENOME “SMART pool” library, Dharmacon/Thermo Scientific) were designed and prepared in 384-well clear bottom ViewPlates (Perkin Elmer) using a protocol for solid phase reverse transfection described elsewhere^44^. An automated liquid handling robot, “MICROLAB STAR” (Hamilton) was used for plate preparation, with each well containing 0.125 μl Lipofectamine 2000 (Life Technologies) and 1.08 pmol of siRNA prior to desiccation. siRNAs targeting the INCENP gene were used as controls for transfection efficiency and a non-targeting siRNA was used as negative control. 72 hours after cell seeding, cells were treated with NPs. siRNA sequences are available in Supporting Information Tables S2 and S4.

### siRNA forward transfection

For the DNM2/CLTC experiments and the validation screens, a classical forward transfection was carried out. On the day before transfection, cells were seeded in clear bottom ViewPlates (Perkin Elmer). Transfection was carried out according to the manufacturer’s instructions, using 0.2 μl or 0.07 μl of Oligofectamine (Invitrogen), and 1.8 pmol or 0.6 pmol of siRNA (Ambion/Life Technologies) per well of a 96- or a 384- well plate respectively. Cells were then incubated for 72 hours (unless otherwise specified) before NP treatment and cell fixation. When cells were transfected with more than one siRNA, the same quantity of each siRNA was used without changing the amount of Oligofectamine. The siRNA sequences for the DNM2 and CLTC experiments are reported in Supporting Information Table S6, while those used for the validation screens are reported in Supporting Information Tables S3 and S5.

### Real time quantitative PCR

Efficiency of mRNA depletion was quantified by real-time quantitative PCR (qPCR). Total RNA from cells treated with siRNA was prepared using an InviTrap Spin Cell RNA Mini Kit (Stratec Molecular). Total RNA was reverse transcribed using a High Capacity cDNA Reverse Transcription Kit (Applied Biosystems), and qPCR analysis was carried out using SYBR green detection in an Applied Biosystems 7500 system, all according to the manufacturers’ instructions. The qPCR cycling conditions were set up as follows: holding stage 50 °C/20 s, 95 °C/10 minute; cycling stage 95 °C/15 s, 60 °C/1 minute (45 cycles). Results were obtained using the −ΔCt method. Primer sequences are listed in Supporting Information Table S7.

### Western blotting analysis

Cells were lysed using RIPA buffer (Thermo Scientific) with complete protease inhibitor cocktail (Roche); for the Clathrin detection experiment, cells were lysed in SDS-PAGE sample buffer. SDS-PAGE was performed on 8–16% TGX pre-cast gels (BioRad) followed by transfer to nitrocellulose membranes. Proteins were detected using rabbit anti-CHC or anti-GAPDH (Cell Signaling), mouse anti-Rab33b (Frontier Institute), rabbit anti-DNM2 or mouse anti-GAPDH (Abcam) primary antibodies followed by incubation with secondary antibodies coupled to either HRP (Invitrogen) or ALKP (Sigma Aldrich). Detection of protein was performed using Western Lightning Plus ECL reagents (Perkin Elmer) or Attophos fluorescent reagent (Roche) and visualized on an LAS-4000 Image Reader (Fuji-film).

### DNA plasmids

The plasmid encoding the human wild-type Rab33b protein fused with GFP was prepared as described previously^45^. Point mutants of Rab33b fused with GFP–Rab33b Q92L and Rab33b T47N and the Rab33b rescue–were prepared using the primers listed in Supporting Information Table S8 using a QuikChange Lightning Site-Directed Mutagenesis Kit (Agilent Technologies), according to the manufacturer’s instructions. DNA sequence analysis confirmed nucleotide changes in the resulting plasmids. GFP-tagged OATL1 was kindly provided by Mitsunori Fukuda^35^. GFP-tagged Myo6 NI wild-type and mutated isoforms were a gift from Folma Buss^41^.

### DNA transfection

The GFP-tagged constructs were transfected into cells using FuGENE6 (Promega) according to the manufacturer’s instructions. For a 24-well plate format, 0.75 μL of FuGENE6 and 0.5 μg of DNA plasmid were used and cells were allowed to express the DNA for 20–22 hours. Cells were then incubated with NPs and fixed as described below. In the rescue experiment, 48 hours following siRNA transfection, cells were transfected with DNA constructs for 22 hours, and then treated with NPs and fixed as described below.

### NP treatment, immunostaining and LysoTracker assay procedures

The NP treatment consisted of incubation of cells with 10 μg/ml of NPs in sf-DMEM for 10 minutes at 37 °C. The NP dispersion was then removed and c-DMEM added for 50 minutes at 37 °C (for 15 minute experiments the incubation with c-DMEM lasted 5 minutes). Cells were fixed with ice-cold methanol (for LAMP1 immunostaining) or 3% paraformaldehyde (for EEA1 immunostaining), stained with Hoechst 33342 (Sigma-Aldrich) and immunostained. Mouse anti-LAMP1 (DSHB, University of Iowa), anti-EEA1 (BD Biosciences), anti-mouse Alexa Fluor 488- and Alexa Fluor 647-conjugated antibodies (Molecular Probes) were used. For GFP-Myo6 transfected cells, the background signal from the soluble GFP-tagged protein was reduced by a 45 second incubation with 0.02% saponin (Sigma-Aldrich) in PBS, before fixation. The GFP signal was enhanced by immunostaining with rabbit anti-GFP primary antibody (custom polyclonal, EMBL-Heidelberg) and Alexa Fluor 488-conjugated secondary antibody. For the lysosomal distribution experiment, cells treated with siRNA for 72 hours were incubated with 250 nM LysoTracker Red (Invitrogen) in c-DMEM for one hour, washed twice with PBS and then fixed with PFA prior to HCS acquisition and analysis.

### Image acquisition and analysis

HCS images were acquired on a fully automated Olympus Scan^R screening microscope with a 40x/0.60 NA LUCPLFLN objective. Images for LAMP1 and NP channels were background corrected using *ImageJ* (National Institutes of Health) using the rolling ball algorithm (radius of 10 pixels) and then analyzed using the Columbus Image Data Storage and Analysis System v2.3.1 (Perkin Elmer). The full image analysis protocol used is available in Supporting Information Table S9. Briefly, using the Hoechst channel, cells were segmented using the “find nuclei” and “find cytoplasm” building blocks. Morphological and intensity measurements were then extracted from the segmented cells in order to remove apoptotic or dividing cells. Cells close to the border of the field of view were also removed from the analysis. LAMP1-positive membranes were detected using the building block called “find spots”. For each well, the software returned the ratio between the average of total intensity of the NP signal associated with LAMP1 and the average of total intensity of the NP signal associated with the cell region (‘LAMP1-associated NP ratio’). Treatments were discarded for lack of viability if less than 50 cells were analyzed per well or less than 150 among the 3 independent experiments. Candidate genes were defined as producing a mild or strong phenotype according to whether the reduction in the LAMP1-associated NP ratio was 2 or 3 standard deviations lower than the average ratio obtained in cells treated with non-targeting siRNA (NEG).

Confocal images were acquired on an FV1000 (Olympus) microscope with a 60x/1.35 NA UPlanSApo oil immersion objective and analyzed using CellProfiler (Broad Institute). The analysis protocol used is fully described in Supporting Information Table S10. Briefly, cell nuclei and cytoplasm were segmented, then the co-localization of NP signal with the different markers was quantified using the Rank Weighted Coefficient (RWC) algorithm^31^ implemented in CellProfiler^46^. Protein-protein interaction network analysis was carried out using the STRING v9.l database selecting human as organism of interest, a medium confidence score (0.400) and allowing 50 additional nodes (http://string-db.org/). All the active prediction methods were selected except for text-mining. Gene family annotation was done using DAVID Bioinformatics Resources v6.7 (http://david.abcc.ncifcrf.gov/). Network layouts were prepared using Cytoscape v3.2.1 selecting the overall score as network edges (http://www.cytoscape.org/). One-tailed Student’s t-tests were performed with Microsoft Excel; one-way ANOVA with Post hoc Tukey test statistical analyses were performed with SPSS Statistics 20 (IBM).

## Additional Information

**How to cite this article**: Panarella, A. *et al*. A systematic High-Content Screening microscopy approach reveals key roles for Rab33b, OATL1 and Myo6 in nanoparticle trafficking in HeLa cells. *Sci. Rep.* **6**, 28865; doi: 10.1038/srep28865 (2016).

## Supplementary Material

Supplementary Information

Supplementary Table 2

Supplementary Table 3

Supplementary Table 4

Supplementary Table 5
