# Supplementary material for: A systematic High-Content Screening microscopy approach reveals key roles for Rab33b, OATL1 and Myo6 in nanoparticle trafficking in HeLa cells
Source: Sci Rep. 2016 Jul 4;6:28865. doi: 10.1038/srep28865 (PMC4931513; doi:10.1038/srep28865)
Supplement: Supplementary Information [file srep28865-s1.doc]

**Supporting Information**

**A systematic High-Content Screening microscopy approach reveals key roles for Rab33b, OATL1 and Myo6 in nanoparticle trafficking in HeLa cells**

Angela Panarella^1,4^, Mariana G. Bexiga^1,3,5^, George Galea^1,3^, Elaine D. O’ Neill^1,6^, Anna Salvati^2,7^, Kenneth A. Dawson^2^ & Jeremy C. Simpson^1,*^

^1^ School of Biology and Environmental Science, University College Dublin, Belfield, Dublin 4, Ireland and Conway Institute for Biomolecular and Biomedical Research, University College Dublin, Belfield, Dublin 4, Ireland.

^2^ Centre for BioNano Interactions, School of Chemistry and Chemical Biology, University College Dublin, Belfield, Dublin 4, Ireland.

^3^ These authors contributed equally to this work.

^4^ Present address: Telethon Institute of Genetics and Medicine, Pozzuoli (Naples), Italy

^5^ Present address: UC-BIOTECH, Center for Neuroscience and Cell Biology (CNC), University of Coimbra, Coimbra, Portugal

^6^ Present address: Randox Laboratories Ltd, Crumlin, Co. Antrim BT29 4QY, UK

^7^ Present address: Groningen Research Institute of Pharmacy, 9713 AV Groningen, The Netherlands

* Correspondence should be addressed to J.C.S. School of Biology and Environmental Science, Science Centre West, University College Dublin, Belfield, Dublin 4, Ireland (jeremy.simpson@ucd.ie)

**Results**

**Table S1. DLS and zeta potential measurements of the nanoparticles used in the study.** DLS and zeta potential results are the average of three separate runs from one representative experiment. Values represent average plus or minus standard deviation between 3 technical replicates.

| **Disp** | **Temp** | **Z-Avg^1^** | **PdI^2^** | **Z-Pot** |
| --- | --- | --- | --- | --- |
|  | °C | nm |  | mV |
| water | 25 | 63.26  ±1.19 | 0.13  ±0.02 | -44.40  ±0.16 |
| PBS | 25 | 66.31  ±0.20 | 0.05  ±0.01 | -35.50  ±0.45 |
| c-DMEM | 25 | 63.51  ±0.57 | 0.1  ±0.01 | -30.63  ±1.93 |
| sf-DMEM | 25 | 61.53  ±0.63 | 0.06  ±0.01 | not measured |
| sf-DMEM | 37 | 56.39  ±0.23 | 0.07  ±0.03 | not measured |

Abbreviations: Disp, dispersant; Temp, temperature; Z-Avg, Zeta-average; PdI, Polydispersity Index; Z-Pot, Zeta potential; sf-DMEM, serum-free DMEM; c-DMEM, complete DMEM.

^1^z-average hydrodynamic diameter extracted by cumulant analysis of the data.

^2^ Polydispersity index from cumulant fitting.


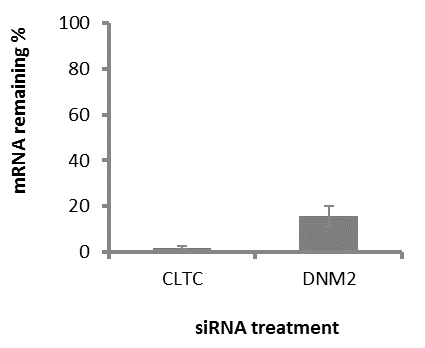


**Figure S1. Quantification of mRNA depletion of endocytosis targets.** Graph showing effectiveness of mRNA downregulation of clathrin heavy chain (CLTC) and dynamin2 (DNM2) as judged by qPCR. Values represent mean and s.e.m. of 3 independent experiments.


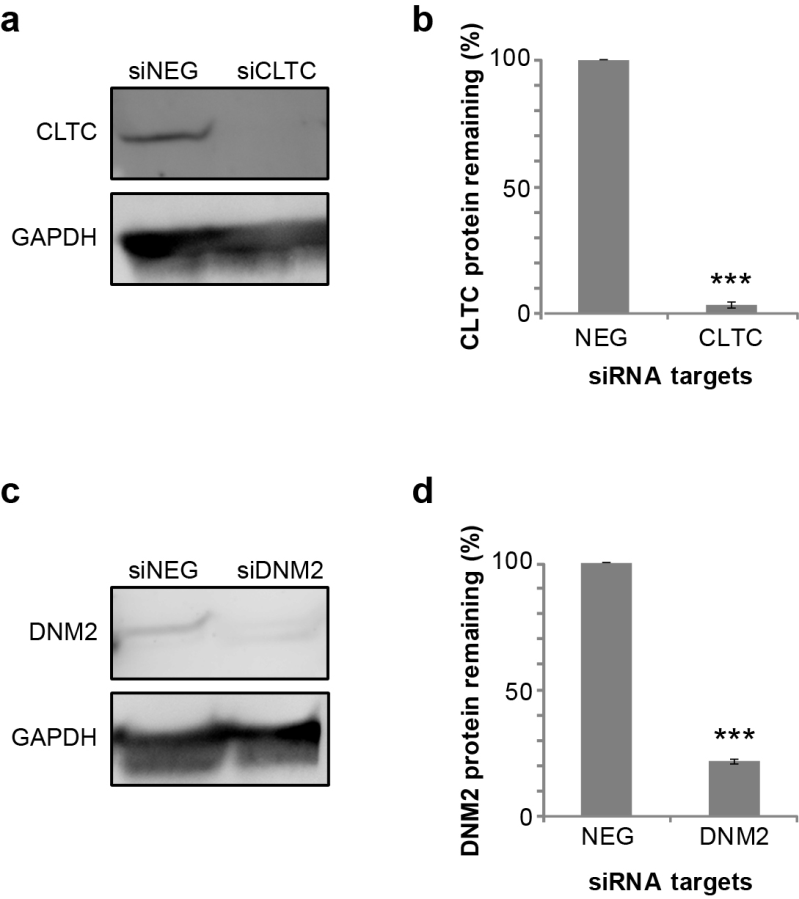


**Figure S2. Quantification of protein depletion of endocytosis targets.** (a, c) Representative images of cell extracts subjected to SDS-PAGE and western blotting and probed for the proteins as indicated. GAPDH is used as a loading control. (b, d) Densitometry analysis of western blots. Values represent mean and s.e.m. of 2 independent experiments, normalized to GAPDH; ***, p-value <0.001 compared to siNEG treated cells.


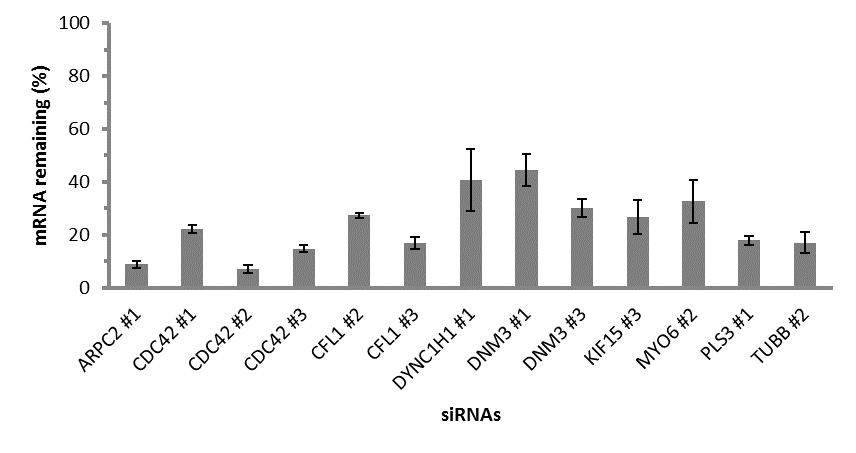


**Figure S3. Quantification of mRNA depletion of cytoskeleton targets.** Graph showing effectiveness of mRNA downregulation of candidate genes as judged by qPCR. The number beside the gene symbol represents which of the 3 independent siRNA molecules was tested. Values represent mean and s.e.m. of 3 independent experiments.


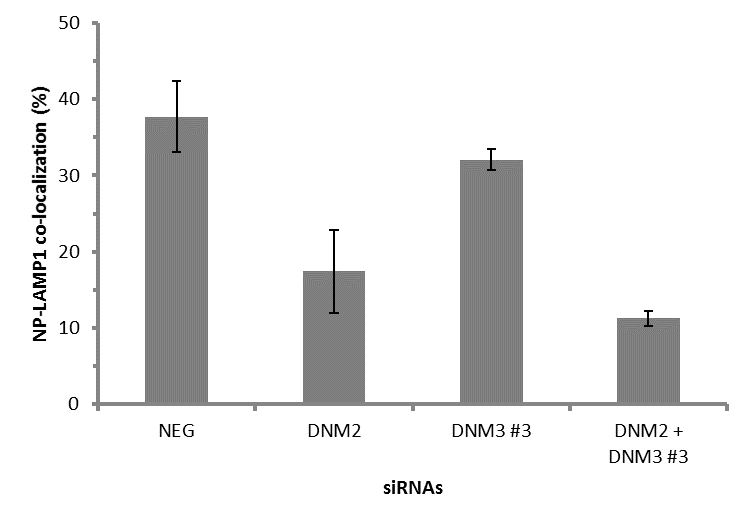


*

**

**Figure S4. Quantification of co-localization of NPs with LAMP1-positive organelles following depletion of various dynamin proteins.** Graphs showing percentage of NPs co-localizing with LAMP1-positive membranes in control cells (NEG) or cells treated with siRNAs targeting DNM2, DNM3 or both as indicated. The number beside the gene symbol represents which of 3 independent siRNA molecules was tested. Values represent mean and s.e.m. of 3 independent experiments; *, p-value < 0.05; **, p-value <0.01 compared to siNEG treated cells.


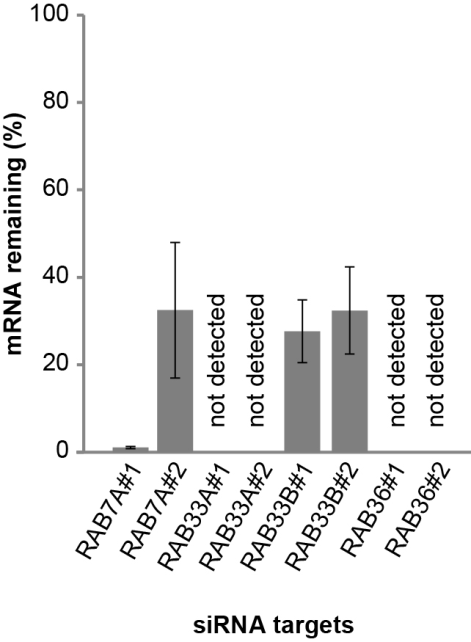


**Figure S5. Quantification of mRNA depletion of Rab targets.** Graph showing effectiveness of mRNA downregulation of candidate genes by two independent siRNAs as judged by qPCR. The number beside the gene symbol represents which of the 2 independent siRNA molecules was tested. Values represent mean and s.e.m. of 3 independent experiments.


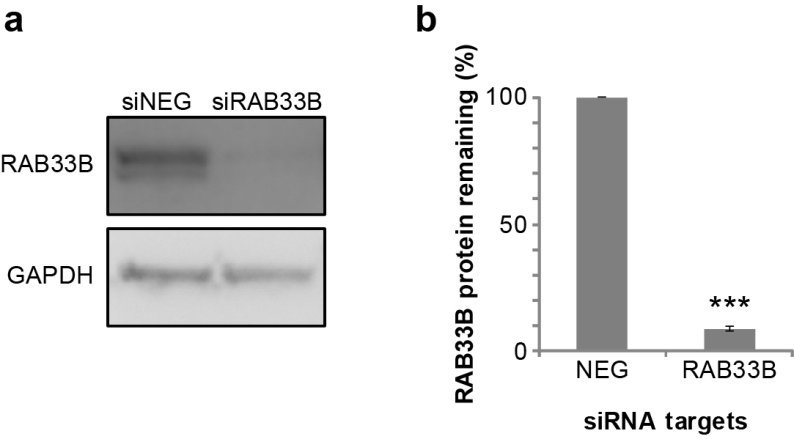


**Figure S6. Quantification of Rab33b protein depletion following siRNA treatment.** (a) Representative images of cell extracts subjected to SDS-PAGE and western blotting and probed for the proteins as indicated. GAPDH is used as a loading control. (b) Densitometry analysis of western blots. Values represent mean and s.e.m. of 2 independent experiments, normalized to GAPDH; ***, p-value <0.001 compared to siNEG treated cells.


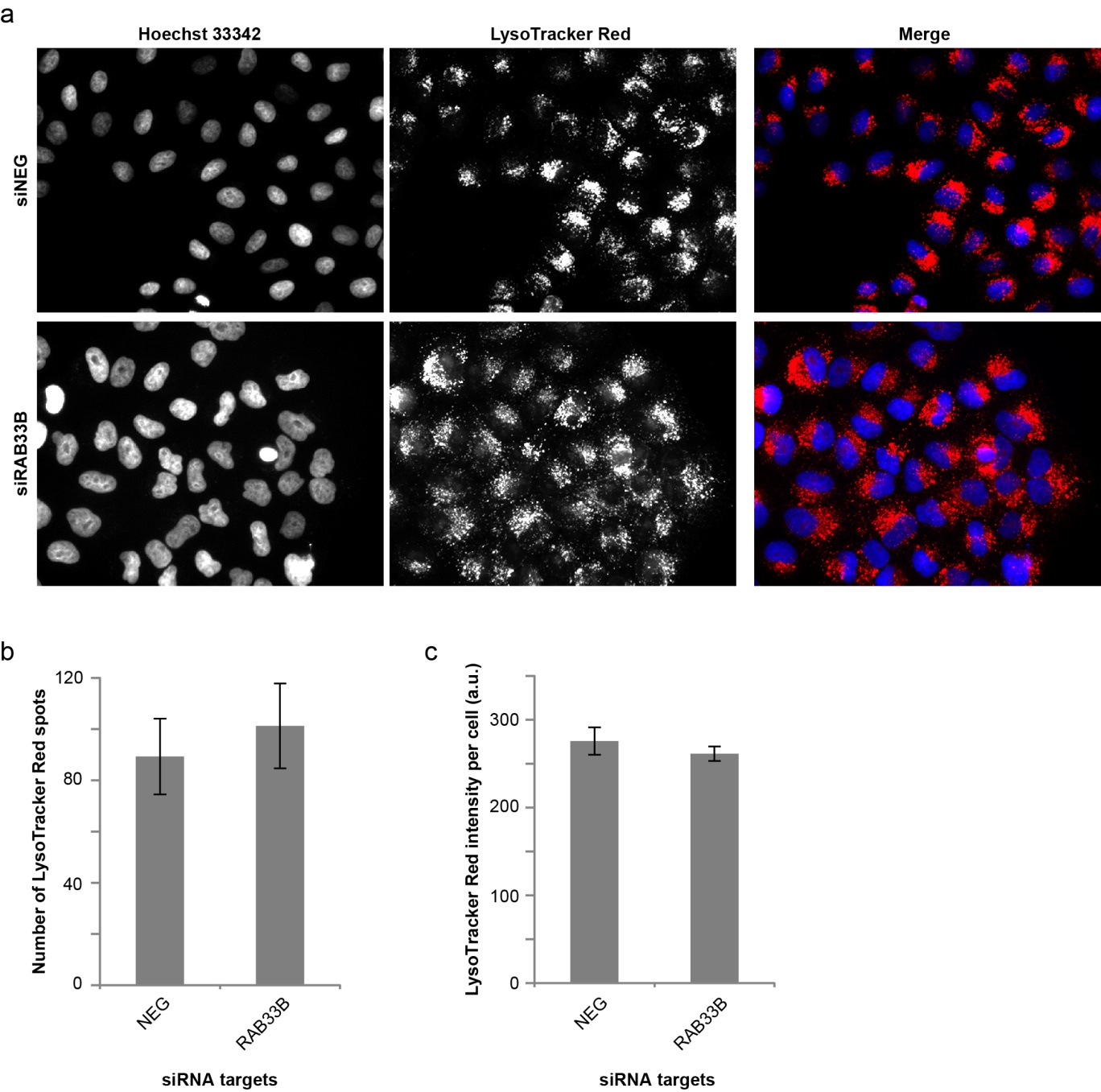


**Figure S7. Quantification of lysosomal distribution in cells depleted for RAB33B.** (a) Representative images of cells treated with LysoTracker for 1 hour after transfection with siNEG or siRAB33B. (b) Graph showing quantification of LysoTracker positive spots and mean LysoTracker Red intensity. Values represent mean and s.e.m. of 3 independent experiments.

**Supporting Information: Materials and Methods**

**Table S6. siRNA sequences for clathrin heavy chain and dynamin2 depletion.**

| **Gene Symbol** | **Gene ID** | **RefSeq Accession Number** | **siRNA ID** | **Sense siRNA Sequence** | **Antisense siRNA Sequence** |
| --- | --- | --- | --- | --- | --- |
| NEG | 0 |  | s813 | UAACGACGCGACGACGUAAtt | UUACGUCGUCGCGUCGUUAtt |
| CLTC | 1213 | NM_004859 | s476 | CGGUUGCUCUUGUUACGGAtt | UCCGUAACAAGAGCAACCGta |
| DNM2 | 1785 | NM_001005360 | s4212 | ACAUCAACACGAACCAUGAtt | UCAUGGUUCGUGUUGAUGUag |

**Table S7. qPCR primer sequences**

| Gene symbol | Forward primer | Reverse primer |
| --- | --- | --- |
| ARPC2 | TTGGAAAGGTGTTCATGCAG | TCCCTGTGGCTAAAGAGGAC |
| CDC42 | CATCGGAATATGTACCGACTGTT | TGCAGTATCAAAAAGTCCAAGAGTA |
| CFL1 | GTGCCCTCTCCTTTTCGTTT | TTGAACACCTTGATGACACCAT |
| DYNC1H1 | AGTTGGTGGAATGTGGGTTG | TGATTGATCTGGGTGATCTGA |
| KIF15 | GCTGCTGAAGCCTATCAGGT | GATGTTGATGCCACACGTCTA |
| MYO6 | CTCCAGCTTCACCCGTACA | CGATCTCCTGTTTCCACTATCC |
| PLS3 | GAAACTTACACCCTTCATCATTCAG | TTCTGCACCAATGTTCACAAC |
| TUBB | ATACCTTGAGGCGAGCAAAA | TCACTGATCACCTCCCAGAAC |
| RAB7 | CAAGATTGACCTCGAAAACAGA | ACGTTGATGGCCTCCTTG |
| RAB33A | GAAATCGAGGGCGAGAAGAT | GTTGCGGTAGTAATGCTCGAC |
| RAB33B | AGAACGAGCGGTGGAGATT | CCATGCTCTTTCTGAATCGTT |
| RAB36 | CACGGCCTTTGACCTCAC | CAGAGCATCCTCCAACCACT |

**Table S8. Primers used for preparation of Rab33b point mutants**

| GFP-Rab33b Q92L | 5’- GGACACAGCAGGACTAGAACGATTCAGAAAGAGCATGG |
| --- | --- |
|  | 5’- CCATGCTCTTTCTGAATCGTTCTAGTCCTGCTGTGTCC |
| GFP- Rab33b T47N | 5’- CCAATGTGGGCAAGAATTGCCTGACCTACCGCTTCTGC |
|  | 5’- GCAGAAGCGGTAGGTCAGGCAATTCTTGCCCACATTGG |
| GFP-Rab33b rescue | 5’- GCAGGACAAGAGAGATTCCGTAAGAGCATGGTTCAGC |
|  | 5’- GCTGAACCATGCTCTTACGGAATCTCTCTTGTCCTGC |

**Table S9. Image analysis routine in Columbus^TM^. Images obtained by HCS automated microscope were analysed with the following routine. The settings used were adjusted to compensate for small variations from plate to plate.**

| **Nuclei segmentation** | |
| --- | --- |
| Channel : | Hoechst |
| Detection method: | B |
| Common Threshold : | 0.4 |
| Area : | > 300 µm² |
| Split Factor : | 5 |
| Individual Threshold : | 0.4 |
| Contrast : | > -0.6 |
| Output population: | Nuclei |
| **Cytoplasm segmentation** | |
| Channel : | Hoechst |
| Method of detection : | D |
| Nuclei : | Nuclei |
| Individual Threshold : | 0.1 |
| **Remove border objects** | |
| Population : | Nuclei |
| Method : | Common Filters |
| Selection : | Remove Border Objects |
| Region : | Cell |
| Output population : | Nuclei selected |
| **Calculate intensity properties (Nuclei)** | |
| Channel : | Hoechst |
| Method of detection: | Standard |
| Population : | Nuclei Selected |
| Region : | Nucleus |
| Measurement : | Mean Intensity |
| Output properties : | Intensity nucleus Hoechst |
| **Calculate morphology properties (Nuclei)** | |
| Population : | Nuclei Selected |
| Method of detection : | Standard |
| Region : | Nucleus |
| Measurement : | Area and Roundness |
| Output properties : | Nucleus morphology |
| **Select population (filtering by nuclear shape and intensity)** | |
| Population : | Nuclei Selected |
| Method : | Filter by Property |
| F1 - Nucleus Roundness : | > 0.75 |
| F2 - Nucleus Area [px²] : | < 16000 |
| F3 - Nucleus Area [px²] : | > 4500 |
| F4 - Intensity Nucleus Hoechst Mean : | < 1200 |
| Boolean Operations : | F1 and F2 and F3 and F4 |
| Output Population : | Nuclei Selected to analyse |
| **Find spots (LAMP1)** | |
| Channel : | green |
| Method of detection: | C |
| Population : | Nuclei Selected to analyse |
| Region : | Cell |
| Radius : | ≥ 7 pixels |
| Contrast : | > 0.16 |
| Uncorrected Spot to Region Intensity : | > 3.8 |
| Distance : | ≥ 1.6 pixels |
| Spot Peak Radius : | 1.6 pixels |
| Measurement: | Calculate Spot Properties |
| Output population: | LAMP1 positive organelles |
| **Calculate intensity properties (NPs)** | |
| Channel : | Red |
| Method of detection: | Standard |
| Population : | Nuclei Selected to analyse |
| Region : | Cell |
| Measurement : | Mean and Sum Intensity |
| Output properties: | Intensity Cell Red |
| **Calculate intensity properties (NPs in LAMP1)** | |
| Channel : | Red |
| Method of detection : | Standard |
| Population : | Nuclei Selected to analyse |
| Region : | LAMP1 positive organelles |
| Measurement : | Mean and sum intensity |
| Output Properties : | Intensity Red in LAMP1 positive organelles |
| **Calculate Properties (LAMP1-associated NP ratio)** | |
| Population : | Nuclei Selected to analyse |
| Method : | By Formula |
| Formula : | A/B |
| Variable A : | Intensity Red in LAMP1 positive organelles Sum |
| Variable B : | Intensity Cell Red Sum |
| Measurement : | Mean and standard deviation |
| Output Property : | LAMP1-associated NP ratio |

**Table S10. Image analysis routine in CellProfiler. Example settings used in the image analysis routine of confocal images to quantify co-localization of NPs with membrane markers (LAMP1 or EEA1).**

| **IdentifyPrimaryObjects** |  |
| --- | --- |
| Input image | Hoechst |
| Name the primary objects identified | Nuclei |
| Typical minimum diameter of objects (pixels) | 60 |
| Typical maximum diameter of objects (pixels) | 150 |
| Discard objects outside the diameter range | Yes |
| Discard objects touching the border of the image | Yes |
| Thresholding method | Otsu adaptive |
| Number of thresholding classes | 2 |
| Minimize the weighted variance |  |
| Threshold correction factor | 1 |
| Lower bound on threshold | 0.05 |
| Higher bound on threshold | 1.0 |
| Method to distinguish clumped objects | Shape |
| Method to draw dividing lines between clumped objects | Shape |
| Automatically calculate size of smoothing filter | Yes |
| Automatically calculate minimum allowed distance between local maxima | Yes |
| Fill holes in identified objects | Yes |
| **IdentifySecondaryObjects** |  |
| Input image | Organelle marker |
| Input objects | Nuclei |
| Name objects identified | Cells |
| Method to identify secondary object | Watershed-Image |
| Thresholding method | Otsu Global |
| Number of thresholding classes | 2 |
| Minimize the weighted variance |  |
| Threshold correction factor | 0.5 |
| Lower bound on threshold | 0.05 |
| Higher bound on threshold | 1.0 |
| Fill holes in identified objects | Yes |
| Discard secondary objects touching the edge of the image | Yes |
| Discard the associated primary objects | Yes |
| Name of new primary objects | FilteredNuclei |
| Retain outlines of the identified secondary objects | Yes |
| Name the outline image | SecondaryOutlines |
| **MeasureObjectSize** |  |
| Objects to measure | Cells and FilteredNuclei |
| **MeasureObjectIntensity** |  |
| Image to measure | Hoechst; organelle marker; GFP |
| Object to measure | FilteredNuclei |
| Image to measure | Organelle marker; GFP;NP |
| Object to measure | Cells |
| **MeasureRWC** |  |
| Image to measure | Organelle marker; NP |
| Measure correlation | Within objects |
| Threshold | 0.15 |
| Object to measure | Cells |
